# Whole exome sequencing reveals genetic landscape associated with left ventricular outflow tract obstruction in Chinese Han population

**DOI:** 10.3389/fgene.2023.1267368

**Published:** 2023-12-18

**Authors:** Zilong Geng, Wenjuan Li, Ping Yang, Shasha Zhang, Shuo Wu, Junhao Xiong, Kun Sun, Dan Zhu, Sun Chen, Bing Zhang

**Affiliations:** ^1^ Key Laboratory of Systems Biomedicine, Ministry of Education, Shanghai Center for Systems Biomedicine, Shanghai Jiao Tong University, Shanghai, China; ^2^ Department of Pediatric Cardiology, Xinhua Hospital, School of Medicine, Shanghai Jiao Tong University, Shanghai, China; ^3^ Shanghai Chest Hospital, Shanghai Jiao Tong University, Shanghai, China

**Keywords:** left ventricular outflow tract obstruction, outflow tract malformation, congenital heart defect, whole-exome sequencing, gene-based burden test

## Abstract

Left ventricular outflow tract obstruction (LVOTO), a major form of outflow tract malformation, accounts for a substantial portion of congenital heart defects (CHDs). Unlike its prevalence, the genetic architecture of LVOTO remains largely unknown. To unveil the genetic mutations and risk genes potentially associated with LVOTO, we enrolled a cohort of 106 LVOTO patients and 100 healthy controls and performed a whole-exome sequencing (WES). 71,430 rare deleterious mutations were found in LVOTO patients. By using gene-based burden testing, we further found 32 candidate genes enriched in LVOTO patient including known pathological genes such as *GATA5* and *GATA6*. Most variants of 32 risk genes occur simultaneously rather exclusively suggesting polygenic inherence of LVOTO and 14 genes out of 32 risk genes interact with previously discovered CHD genes. Single cell RNA-seq further revealed dynamic expressions of *GATA5*, *GATA6*, *FOXD3* and *MYO6* in endocardium and neural crest lineage indicating the mutations of these genes lead to LVOTO possibly through different lineages. These findings uncover the genetic architecture of LVOTO which advances the current understanding of LVOTO genetics.

## Introduction

Cardiac outflow tract (OFT) abnormalities characterized by an array of structural deviations within the region responsible for facilitating the egress of blood from the heart account for approximately 30% of CHD patients (Srivastava and Olson, 2000). One important category of OFT anomalies is left ventricular outflow tract obstruction (LVOTO), with the stenotic lesions at the region of left ventricular outflow tract (could be valvular, sub-valvular or supravalvular). These stenoses can occur alone or in combination, obstruct the blood flow and induce the overload of left ventricle, if untreated which results in ventricle hypertrophy and systolic failure eventually. The majority of LVOTOs are congenital and account for around 6% of CHD (Gaynor and Elliott, 1993). The congenital LVOTO encompasses a range of conditions, including aortic stenosis (AS), bicuspid aortic valve (BAV), coarctation of the aorta (CoA), and anomalous subaortic stenosis (ASA).

Aortic stenosis represents 3%–6% of CHDs with features of narrowed aortic valve orifice and curbed blood flow from left ventricle to aorta. AS is an important cause of congestive heart failure in neonates and fetal demise (Minners et al., 2020). Bicuspid aortic valve is the most common congenital aortic valve malformation with the presence of only two valve leaflets instead of the normally three. Clinical manifestations of BAV can vary from mild to severe. It is the leading cause of AS in patients under 70 years old though might with subtle symptoms in early years (Pujari and Agasthi, 2023). Coarctation of the aorta accounts for 6%–8% of CHDs, and is often accompanied by BAV (∼60%) (Sinning et al., 2018), aortic arch hypoplasia (∼18%) (Ma et al., 2017), ventricular septal defect (∼13%) and other congenital heart defects.

Genetic mutations had been discovered to play a large role in LVOTO etiology. Previous studies have identified *NOTCH1* mutations (p.Arg1108Ter and p.His1505del) in patients with AS and early BAV (Garg et al., 2005). Genetic variants of Cardiac GATA transcription factor *GATA4*, *GATA5*, *GATA6* and *TBX5* were also found to be related to BAV in multiple studies (Bravo-Jaimes and Prakash, 2020; Jiang et al., 2020). A linkage analysis based on family pedigrees reported associations between AS and CoA in chromosomes 2p23, 10q21, and 16p12 (McBride et al., 2009). These studies suggested that there was a shared genetic mechanism across divergent syndromes of LVOTO. Although substantial efforts have been made to uncover LVOTO genetic variants, understanding of the inherent genetic status and mechanism remained largely incomplete especially for the Asian population.

To explore the genetic inherent mechanisms underlying LVOTO of Chinese CHD patients, we performed whole-exome sequencing (WES) analysis on 106 CHD patients with LVOTOs. We discovered rare deleterious variants including missense SNV, microdeletion and insertion in LVOTO patients which are higher than in control subjects. By gene-based burden testing, we identified 32 candidate genes, which were significantly enriched in transcriptional regulation, cardiac development, and neural development. Among 32 candidate risk genes, 14 were found to form an interacting network with known OFT malformation-related genes. Utilizing scRNA-seq and scATAC-seq, we found *GATA5* and *GATA6* may play essential roles in endocardial-derived OFT lineage, while *FOXD3*, *MYO6* and *GATA6* in neural crest-derived lineages. In conclusion, our study uncovers a genetic architecture associated with LVOTO in the Chinese population and offers insights into the pathogenic underpinnings of this complex congenital defect.

## Materials and methods

### Study cohort

Our study enrolled 106 individuals from Han Chinese population diagnosed with LVOTO with clinic syndrome: atrial septal defect (ASD) and ventricular septal defect (VSD). 100 healthy individuals who were ruled out CHD and other birth defects were recruited to the cohort as control. We excluded participants with known chromosomal or syndromic disorders to reduce the statistical errors caused by other confounding factors. This study was approved by the Ethics Committee of Xinhua Hospital Affiliated to Shanghai Jiao Tong University School of Medicine, and conducted in accordance with the principles outlined in the Declaration of Helsinki. All participants or their legal guardians provided written informed consent before being enrolled in the study.

### Whole exome sequencing (WES)

Blood samples were obtained, and genomic DNA was subsequently extracted employing the QIAamp™ DNA Blood Mini kit (Qiagen) in strict accordance with the manufacturer’s recommended protocol. Whole-exome sequencing (WES) was performed utilizing the Agilent SureSelect Target Enrichment kit (V6 58 Mb; Agilent Technologies) for sequence capture, followed by sequencing on the Illumina HiSeq2500 platform. The sequencing aimed to achieve a target depth of 100 fold coverage to ensure accuracy.

### Variants calling

To obtain high-quality sequencing data for downstream analysis we used the Genome Analysis Toolkit (GATK) best practice to process the fastq file (McKenna et al., 2010). Firstly, the raw sequencing reads were aligned to the hg19 reference genome using the Burrows-Wheeler Aligner (BWA) (Li and Durbin, 2009). Subsequently, we identified and marked PCR duplicates using the MarkDuplicates tool in GATK. To recalibrate the base quality scores of the aligned reads, we used Base Quality Score Recalibration (BQSR) with the BaseRecalibrator. Next, variant calling was performed using the HaplotypeCaller tool in GATK v4.1.9.0 with default parameters. To ensure that the variant calls were accurate, we performed Variant Quality Score Recalibration (VQSR) using the VariantRecalibrator and ApplyVQSR tools in GATK. Finally, HaplotypeCaller was used to call variants.

### Quality control and population stratification analysis

To eliminate potential confounding factors due to differences in genetic ancestry among study participants, we performed population stratification analysis on the cohort. We merged the 1000 Genomes Project (1KGP) reference dataset (Clarke et al., 2012) with the data from our cohort. We then performed PCA on the merged dataset using PLINK (Purcell et al., 2007). We plotted the first two principal components (PC1 and PC2) of the PCA results to visualize the genetic structure and population stratification in our cohort and the 1KGP reference panel.

### Annotation and filtering of rare and deleterious variants

In this study, we aimed to investigate the role of rare and deleterious variants enriched in the LVOTO patients. To this end, we utilized ANNOVAR (Wang et al., 2010) to annotate the variants. Population frequency databases, Genome Aggregation Database (gnomAD v2.1.1) (Koch, 2020) and Exome Aggregation Consortium (ExAC) were used to assess the frequency of the variants in the general population. In this study, rare variants are defined as having a minor allele frequency (MAF) of less than 1% in both the gnomAD and ExAC databases. Next, to assess the potential functional impact of the identified rare variants, we employed multiple *in silico* prediction algorithms, including Sorting Intolerant From Tolerant (SIFT), Polymorphism Phenotyping v2 (PolyPhen2), MutationTaster and Combined Annotation Dependent Depletion (CADD). All database downloads were conducted following the protocol provided by the ANNOVAR website (https://annovar.openbioinformatics.org/en/latest/user-guide/). Variants were classified as deleterious if they were predicted to be damaging or deleterious by at least two of the four algorithms. Finally, rare deleterious variants were kept to perform burden test.

### Gene based burden test

Gene based burden test was performed to evaluate the association between the identified rare deleterious variants and the LVOTO phenotype using RVTESTS (Zhan et al., 2016). Then we applied Bonferroni correction on the *p*-value of burden test to account for multiple testing. Finally, genes with adjusted *p*-value less than 0.05 were defined as candidate genes enriched with rare deleterious variants.

### Gene ontology enrichment analysis

We carried out functional annotation using the Database for Annotation, Visualization, and Integrated Discovery (DAVID; https://david.ncifcrf.gov) to perform Gene Ontology (GO) term analyses. The GO terms are focused on biological processes to annotate potential function of the gene set.

### Protein protein interaction network analysis

To investigate the patterns of occurrence of candidate genes in patients, we employed the somaticInteractions function from maftools to conduct co-occurrence statistics between the genes. This method is based on the pair-wise Fisher’s exact test as detailed in the maftools documentation (https://github.com/PoisonAlien/maftools). Then we used the CHDgenes database (http://chdgene.victorchang.edu.au/) to identify known OFT malformation-related genes. Then we investigate the protein-protein interaction (PPI) network using the STRING database (version 11.0; https://string-db.org/). We set the confidence score threshold at 0.7 to ensure a high-quality interaction network and to minimize the inclusion of false-positive interactions. Cytoscape (version 3.9.0; https://cytoscape.org/) was employed for the visualization and analysis of the protein-protein interaction (PPI) network. To identify hub genes within the PPI network, we utilized the cytoHubba plugin based on the PPI topological features.

### Single cell RNA-seq and single cell ATAC-seq analysis

The processed single-cell RNA-seq and single-cell ATAC-seq datasets were acquired from GSE181346 and https://doi.org/10.5281/zenodo.7063223 respectively (Ameen et al., 2022). The scRNA-seq dataset had been subjected to quality control and preprocessing steps including read alignment, gene assignment for scRNA-seq. We utilized the Seurat package (version 4.0; https://satijalab.org/seurat/) in R and scanpy (Wolf et al., 2018) to conduct downstream analysis, such as normalization and visualization of single-cell RNA sequencing data. Pseudotemporal analysis was conducted using Monocle3 (http://cole-trapnell-lab.github.io/monocle3/) and CellRank (Lange et al., 2022). For the ATAC-seq datasets, cell type-specific marker peaks across various cell populations were identified using the ArchR package (Granja et al., 2021). To perform peak calling in each cell clusters, we used MACS2 (Zhang et al., 2008) for scATAC-seq datasets. Peaks were associated with genes based on the annotations provided in the dataset.

## Results

### LVOTO cohort

A cohort of 106 LVOTO probands, 69 males (65.09%), 37 females (34.91%) and 100 health controls were enrolled from 2016 to 2023. 82 LVOTO patients (77.36% of total) were at age less than 1 year, 18 patients (16.98%) were at age of 1–3 years and six patients (5.66%) older than 3 years (Table 1). All patients were diagnosed with LVOTO, including BAV (8.49%, *n* = 9), AS (46.23%, *n* = 49), and CoA (49.06%, *n* = 52) (Table 2). Notably, AS and CoA co-occurred in four cases. Several other cardiac abnormalities were observed: 45 atrial septal defects (ASD, 42.45%), 42 ventricular septal defects (VSD, 39.62%), 35 patent ductus arteriosus (PDA, 33.02%) and five mitral stenosis/mitral regurgitation (MS/MR, 4.72%). This was consistent with previous reports that LVOTO is usually complicated with multiple pathological phenotypes outside OFT. The control group consisted of 100 healthy children, confirmed not to have any developmental anomalies.

**TABLE 1:** Demographic characteristics of LVOTO patients.

Number of patients (%)	
Sex	
Male	69 (65.09%)
Female	37 (34.91%)
Age	
<1 year	82 (77.36%)
1–3 years	18 (16.98%)
>3 years	6 (5.66%)

**TABLE 2:** Diagnosis of the patients with LVOTO.

Number of patients (%)	
Phenotype	
BAV	9 (8.49%)
AS	49 (46.23%)
CoA	52 (49.06%)
Combined cardiac malformations	
Atrial Septal Defect	45 (42.45.6%)
Ventricular Septal Defect	42 (39.62%)
Patent Ductus Arteriosus	35 (33.02%)
Mitral Stenosis/Mitral Regurgitation	5 (4.72%)
Complications	
Pulmonary Hypertension	42 (39.62%)
Heart Failure	3 (2.83%)

### Identification and analysis of single nucleotide variants in the LVOTO cohorts

We applied the Genome Analysis Toolkit (GATK) Best Practices workflow to analyze the whole-exome sequencing data and found a total of 271,462 high-confidence single nucleotide variants (SNVs) and indels in the LVOTO probands. On average, each proband harbored 39,057 Single Nucleotide Variants (SNVs) and 5,597 indels including insertions or deletions, which was significantly higher than the control group with average of 38,764 SNVs and 4,226 indels per subject (Supplementary Figure S1).

**FIGURE 1:**
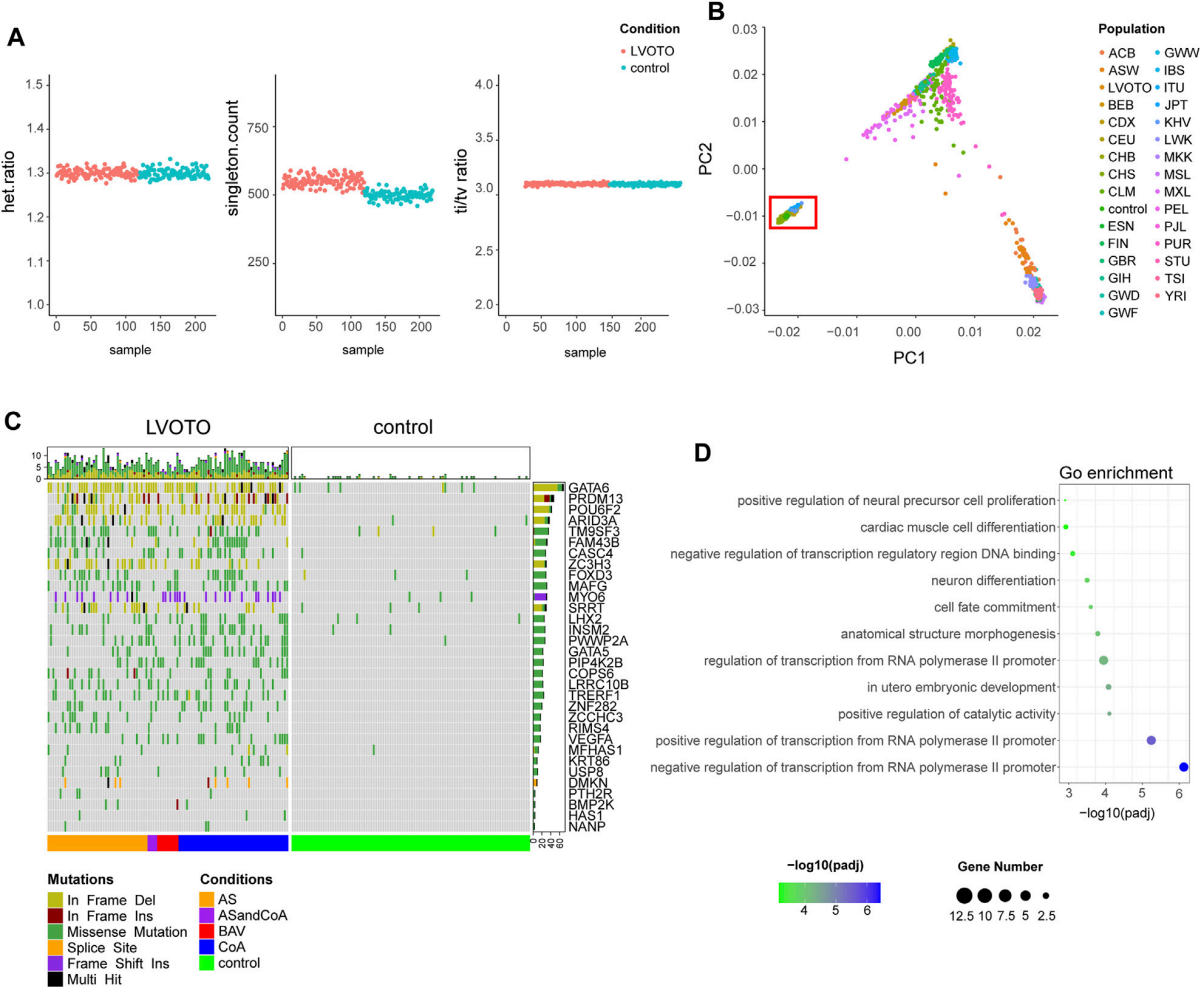
Identification of candidate genes with rare deleterious variants. **(A)** Quality control of variants in LVOTO patients and controls, including heterozygous mutation rate, singleton statistics, and transition-to-transversion ratio. **(B)** Population stratification comparison with the 1000 Genomes Project. The red box indicates the LVOTO patients, control subjects, and Chinese samples from the 1000 Genomes Project. **(C)** Landscape of rare deleterious variants in LVOTO-associated candidate genes based on burden test. **(D)** Functional enrichment analysis of the candidate gene set.

To ensure the quality of our analysis, we evaluated the heterozygosity rate, the number of singletons and the ratio of transitions to transversions (Ti/Tv) (Figure 1A). The heterozygous to non-heterozygous ratio in our cohort was 1.3, the transition to transversion ratio was 3.11, which were in agreement to standard expectations for whole-exome sequencing (Wang et al., 2015). The number of singletons averaged at 517 per sample and no apparent outliers were observed. We also performed population stratification analysis by integrating our dataset to 1000 Genomes Project (1KGP) reference panel and found a complete overlap with Chinese Han population, which ruled out the confounding effect from population stratification (Figure 1B).

Compared with common diseases, LVOTO is rare and prone to be caused by rare variants. Therefore, we focused on the rare variants. To refine our identification and on potentially pathogenic variants, we adhered to the guideline provided by the American College of Medical Genetics and Genomics (ACMG). By leveraging GATK and ANNOVAR platform, we identified 106,476 rare deleterious variants with minor allele frequency (MAF) less than 1% in both the Genome Aggregation Database (gnomAD) and the Exome Aggregation Consortium (ExAC) population databases. 71,430 rare deleterious variants were discovered in LVOTO patient cohort, with each patient carrying an average of 4,600 rare deleterious SNVs and 301 indels. In contrast, the control group exhibited 52,989 such variants, with each individual carrying an average 4,477 rare deleterious SNVs and 265 indels (Supplementary Figure S1). This disparity suggested the potential contribution of these rare variants to the pathophysiology of LVOTO.

### LVOTO-associated candidate genes identified by gene-based burden test

In order to identify genes enriched with rare deleterious SNVs and indels in LVOTO patients, we performed a gene-based burden test using RVTESTS program. We defined LVOTO risk genes as those with *p*-value less than 0.05 adjusted by multiple testing correction. As a result, 32 candidate genes with 572 rare deleterious variants in LVOTO patients *versus* 32 in controls were potentially associated with LOVTO pathogenesis (Figure 1C, Supplementary Tables S1, S2). The variants of *GATA6*, *PRDM13*, *POU6F2*, *ARID3A*, *ZC3H3* and *SRRT* were predominantly in a form of frame deletion, in *MYO6* were frameshift insertions, and in the rest were the missense mutations. Functional enrichment analysis indicated these 32 genes are involved in transcription regulation, embryonic development and cardiac muscle cell differentiation (Figure 1D). Interestingly, neural cell differentiation term was also enriched, indicating some of LVOTO risky genes were from neural crest lineage that plays an important role in OFT septation and aortic arch patterning.

Among 32 candidate genes, *GATA5* (*p* = 0.0096 in burden test) and *GATA6* (*p* = 6.22E-10 in burden test) had been known to be associated with LVOTO. *GATA5* and GATA6 are the zinc finger transcription factors specifically binding to GATA DNA motif and play critical roles in regulating early cardiac gene expression and cardiac cell differentiation. Previous human genetics study showed that *GATA5* and *GATA6* were associated with BAV and AS (Shi et al., 2014; Bravo-Jaimes and Prakash, 2020). Animal model study illustrated that loss of *GATA5* in mice resulted in BAV (Laforest et al., 2011). Herein, we identified a c.40G>C missense mutation of *GATA5* in 22 out of 106 patients, which resulted in alanine to proline change at position 14 (p.Ala14Pro) in a N terminal disordered region. Among these 22 patients, 17 had CoA combined with VSD and AVSD and five patients had AS. This variant had not been reported before to be associated with outflow tract malformation in western population. Same mutation observed in a substantial portion of our small cohort indicated c.40G>C might be a founder mutation specifically affected Chinese Han people. We also found a heterozygous c.993_998 microdeletion of *GATA6* which causes the deletion of Histidine at position 332 and 333 (p.His332_His333del) in a disordered region (Supplementary Figure S2). Similar to *GATA5*, 58 patients harbored this deletion, indicating it may be a founder variant in Chinese Han population as well.

*VEGFA* (Vascular Endothelial Growth Factor-A) is an essential member of the vascular endothelial growth factor (VEGF) family. Nonsense mutations in *VEGFA* have been discerned in patients diagnosed with LVOTO (Zhao et al., 2010). Lack of OFT septation is seen in *Vegfa* ^120/120^ mouse model expressing a short form of *VEGFA* 120 (Plein et al., 2015). Several pathways such as *BMP* and *SEMA3C* regulate OFT development by interacting with *VEGFA*. Here, we found *VEGFA* is enriched in LVOTO patient cohorts as 17 variants were discovered in LVOTO *versus* none in controls (*p* = 0.041). Among them, a c.329G>T missense mutation causing arginine to leucine substitution at position 139 (p.Arg110Leu) were found in 15 patients. Intriguingly, within this cohort, 11 patients were diagnosed with CoA, 3 with BAV, and one presented with a combination of CoA and AS. Interestingly, none with AS only. These well-recognized candidate genes association with LVOTO, certified the effectiveness of our gene-based burden testing approach.

### Molecular network of LVOTO candidate genes

**FIGURE 2:**
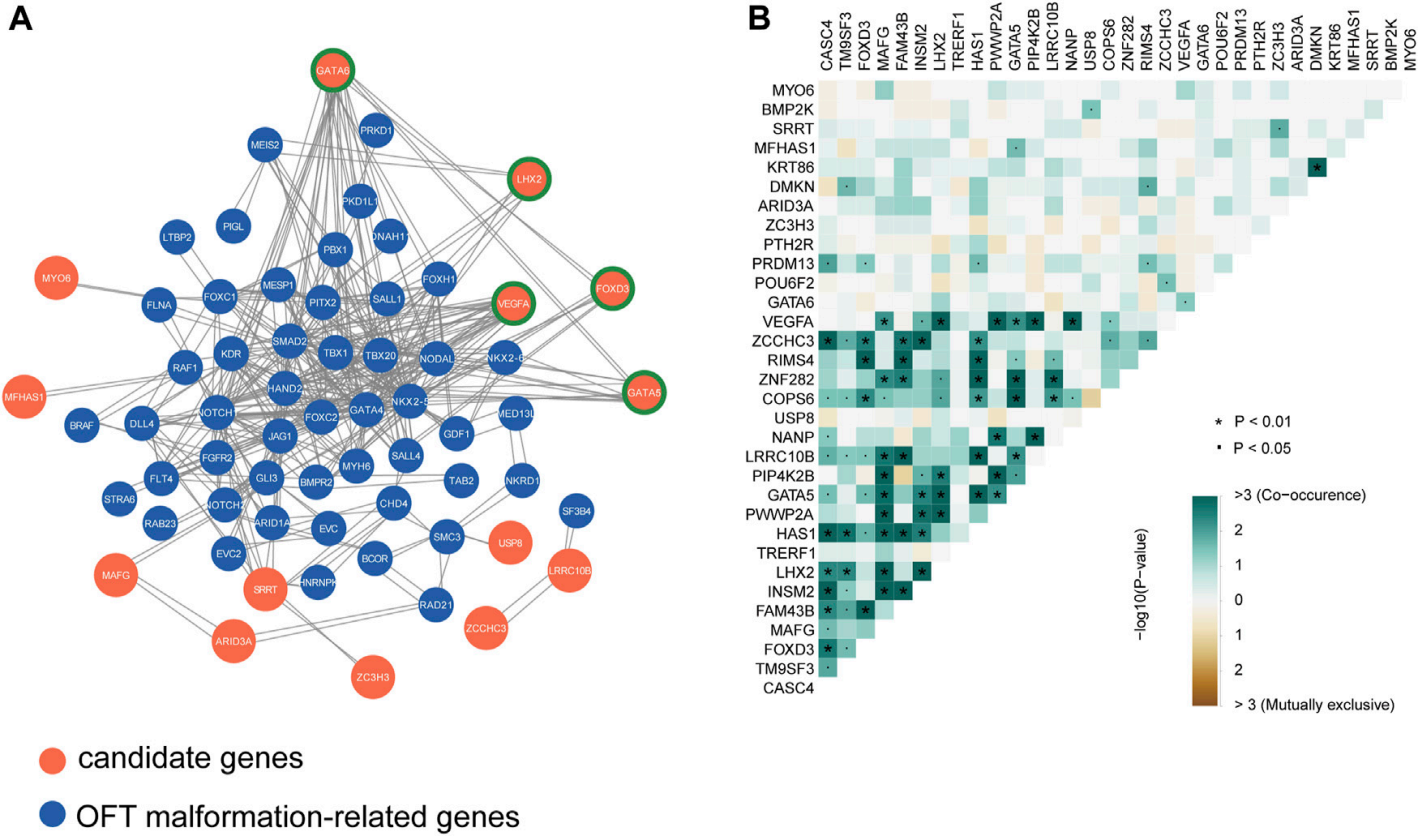
Interactions analysis among candidate genes and known CHD genes. **(A)**Protein-protein interaction network of candidate genes and known OFT abnormality risk genes, highlighting the potential functional relationships and molecular pathways shared by these genes in the context of outflow tract development and dysfunction. Node genes are marked with a green border. **(B)** Co-occurrence statistics of LVOTO-associated candidate genes, demonstrating the co-occurrence of these genes in patients with LVOTO.

For complex diseases, the genetic risk genes usually do not exert their roles alone but in concert with other genes in a regulatory network. Therefore, we conducted a protein-protein interaction network analysis using STRING to assess the interaction between our candidate genes and known disease-causal genes of outflow tract malformations and obstructive lesions quoted from the congenital heart disease risk gene database (http://chdgene.victorchang.edu.au/, Supplementary Table S3). 49 OFT risky genes including *NOTCH1*, *GATA4* and *NKX2-5* were found to be connected with 14 LVOTO risk genes (Figure 2A). Among them, we identified five key hub genes including *GATA5*, *GATA6* and *VEGFA*. These genes demonstrated the high degree of connections, interacted with multiple quoted CHD genes, including *NKX2-5*, *HAND2*, and *GATA4*.

*FOXD3*, a member of the forkhead box (FOX) family transcription factors, also had pleiotropic connections with *GATA6*, *NKX2-5* and *SALL4*. *FOXD3* is expressed in cardiac neural crest cells (Stankunas et al., 2010). These cells migrate and contribute to the formation and remodeling of the pharyngeal arch arteries and the septation of the cardiac outflow tract (Yamagishi, 2021). Deletion of *Foxd3* in zebrafish results in significant losses of distinct neural crest derivatives. A subset of *Foxd3* mutant mouse exhibited severe cardiac neural crest defects and absence of the outflow tract septation (Teng et al., 2008). In our cohort, *FOXD3* rare deleterious variants were highly enriched in LVOTO patients (*p* < 0.05 in burden test). Among these variants, we discovered a previously unreported missense mutation c.803C>G in 23 LVOTO patients, which resulted in an alanine to glycine substitution at position 268 (p.Ala268Gly). Importantly, this mutation was not observed in our control group. This evidence suggested that *FOXD3* might be a LVOTO-causal gene in Chinese population.

*LHX2* is another gene in our 32 candidates gene list interacting with known CHD genes. *LHX2* is a LIM domain-containing transcription factor specifically involving in pharyngeal mesoderm development. Knockout of this gene in mouse results in pharyngeal muscle defects, as well as DiGeorge syndrome-like phenotypes such as variety of outflow tract malformations and tetralogy of Fallot (Harel et al., 2012). In our cohort, we discovered a missense mutation c.92C>T in *LHX2* in 22 individuals. This mutation results in an amino acid substitution from serine to phenylalanine at position 31 (p.Ser31Phe).

The candidate genes may have two distinct modes of action in pathogenesis: the first, with minor pathogenicity, and simultaneous occurrence in different probands; the second, with strong pathogenicity, mutually exclusive occurrence in probands (Fahed et al., 2013). Therefore, we conducted a concurrency analysis of rare deleterious variants in 32 risky genes to reveal their genetic behaviors in LVOTO (Figure 2B). The results demonstrated that compared to mutual exclusion, much more concurrency (>3) was observed between 32 genes (*p* < 0.01) indicating a polygenic inheritance in LVOTO pathogenesis. Notably, we found strong co-occurrence of *GATA5* with *LHX2* and *VEGFA* with *LHX2* in LVOTO, suggesting a synergetic role of *LHX2* with *VEGFA* and *GATA5* in LVOTO pathogenesis.

### LVOTO-associated genes in endocardial lineage

**FIGURE 3:**
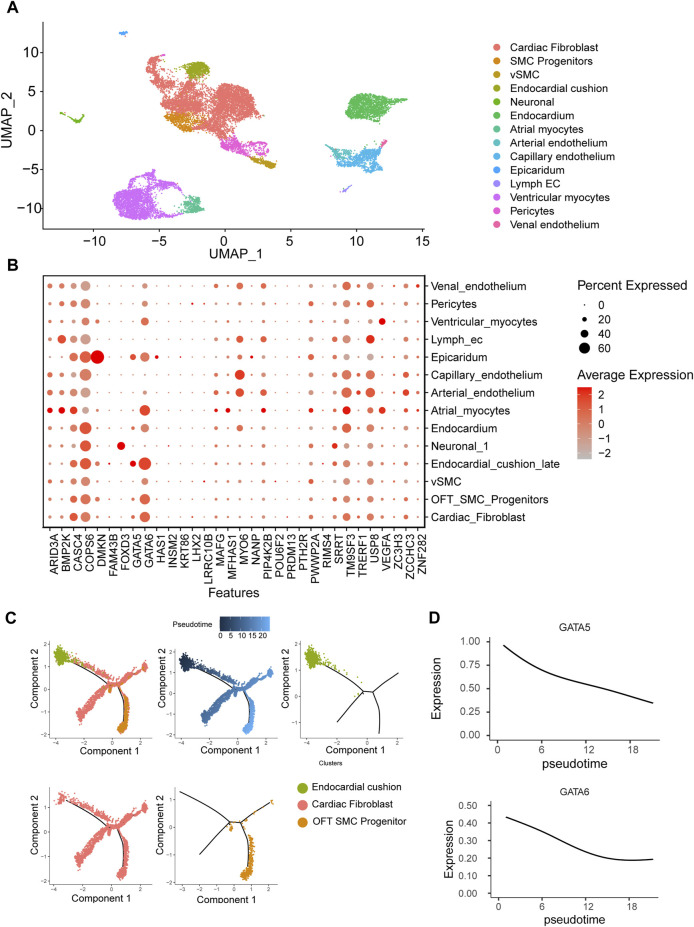
Human embryonic heart single-cell transcriptome analysis. **(A)** Clustering of human embryonic heart single cells. **(B)** Expression patterns of candidate genes across distinct cell populations. **(C)** Pseudotemporal analysis of endothelial-to-mesenchymal transition process of endocardial cushion cells. **(D)** Dynamic expression patterns of *GATA5* and *GATA6* along the pseudotime trajectory.

To gain insight into the spatiotemporal expression profile of the candidate genes, we analyzed the previously published single-cell transcriptomic dataset of human embryonic cardiac development at 6PCW (post-conception weeks), 8PCW, 12PCW and 18PCW (Ameen et al., 2022). The cells were grouped into 14 clusters by using UMAP dimensionality reduction method. Major cell types including myocytes, fibroblast, endothelium, smooth muscle and endocardium were all obtained (Figure 3A). As reported in mouse studies, *GATA6* was highly expressed in endocardium, myocardium, smooth muscle progenitor and fibroblast, while *GATA5* expression is much limited to endocardium and epicardium (Laforest and Nemer, 2011). *FOXD3* was specifically expressed in neural crest cells while *DMKN* more specifically in epicardium. There were several genes such as *CASC4*, *TM9SF3*, *USP8* and *COPS6* ubiquitously expressed in all tissues, but their functions in OFT remain ambiguous (Figure 3B).

**FIGURE 4:**
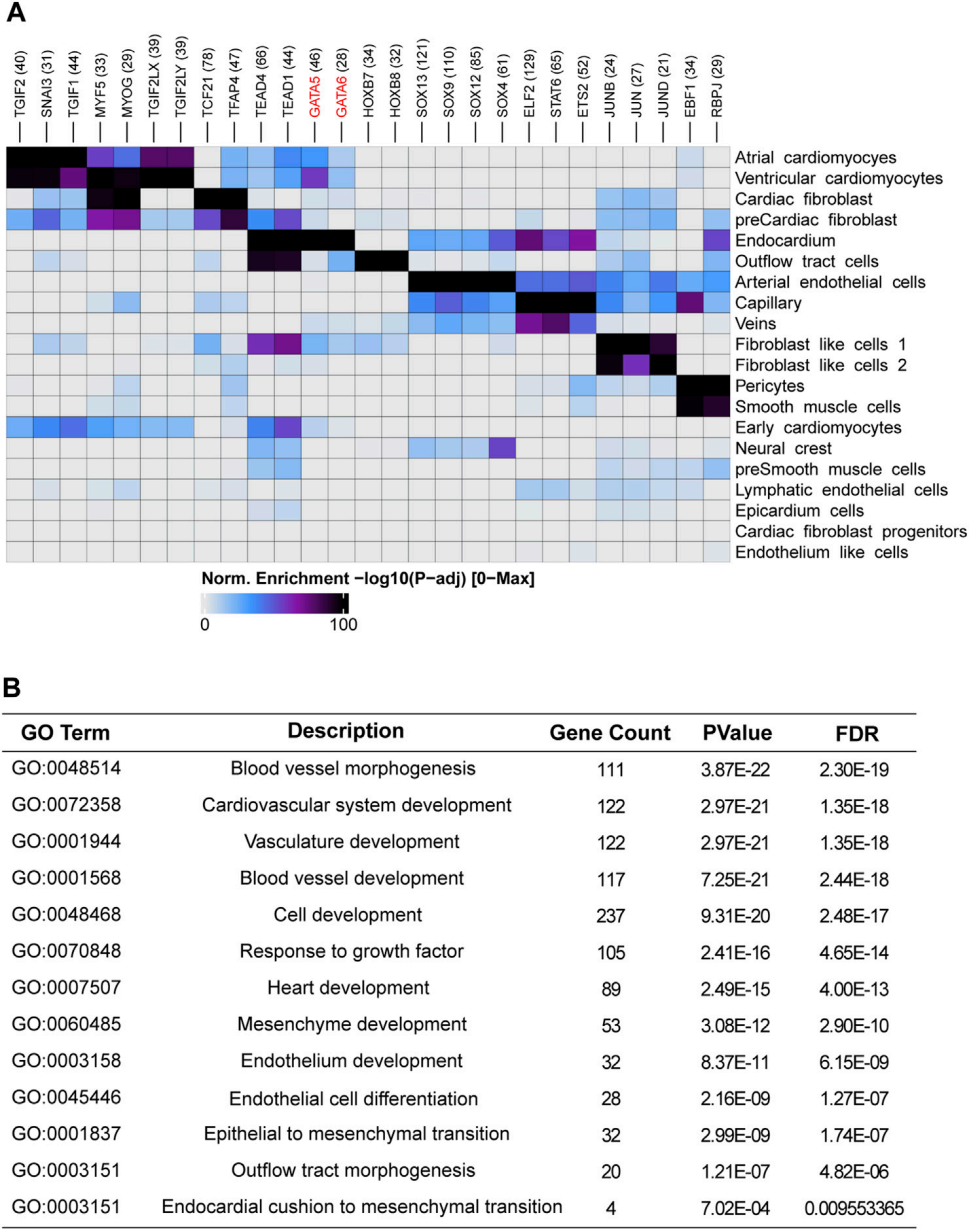
Single-cell accessibility analysis of the human embryonic heart. **(A)** Transcription factor motif enrichment analysis in cell-type-specific accessible genomic regions for different cell populations within the human embryonic heart. **(B)** Functional enrichment analysis of *GATA5* and *GATA6* potential target genes in endocardium.

One of major cell source for OFT is endocardium-derived cells through endothelial-to-mesenchymal transition (EndMT) that contributes to mesenchymal valves and OFT cushion (Ma et al., 2005). Interestingly, scRNA-seq uncovered two isolated populations of endocardium. One is in spatial proximity to myocardial fibroblast cells and outflow tract smooth muscle progenitor clusters, suggesting a close transcriptomic relationship among them. We conducted a pseudotime analysis and observed a decreasing expression of *GATA5* and *GATA6* from endocardium to fibroblast cells (Figures 3C, D), indicating the roles of *GATA5* and *GATA6* in the EndMT process. To further elucidate the genomic binding profiles and downstream effectors of *GATA5* and *GATA6* in human endocardial lineage, we analyzed scATAC-seq dataset (GSE181346) for human cardiac development (Ameen et al., 2022). Using MACS2, we called peaks for each cell population, obtaining a total of 2,352 peaks in endocardial lineage. We identified a high prevalence of *GATA5* and *GATA6* (Figure 4A) by motif enrichment analysis. GO term analysis of *GATA5* and *GATA6* motif-associated genes included outflow tract morphogenesis, endocardial cushion to mesenchymal transition, indicating *GATA5* and *GATA6* regulated the expression of these genes that contributed to endocardial lineage and OFT development (Figure 4B).

### LVOTO-associated genes in neural crest lineage

**FIGURE 5:**
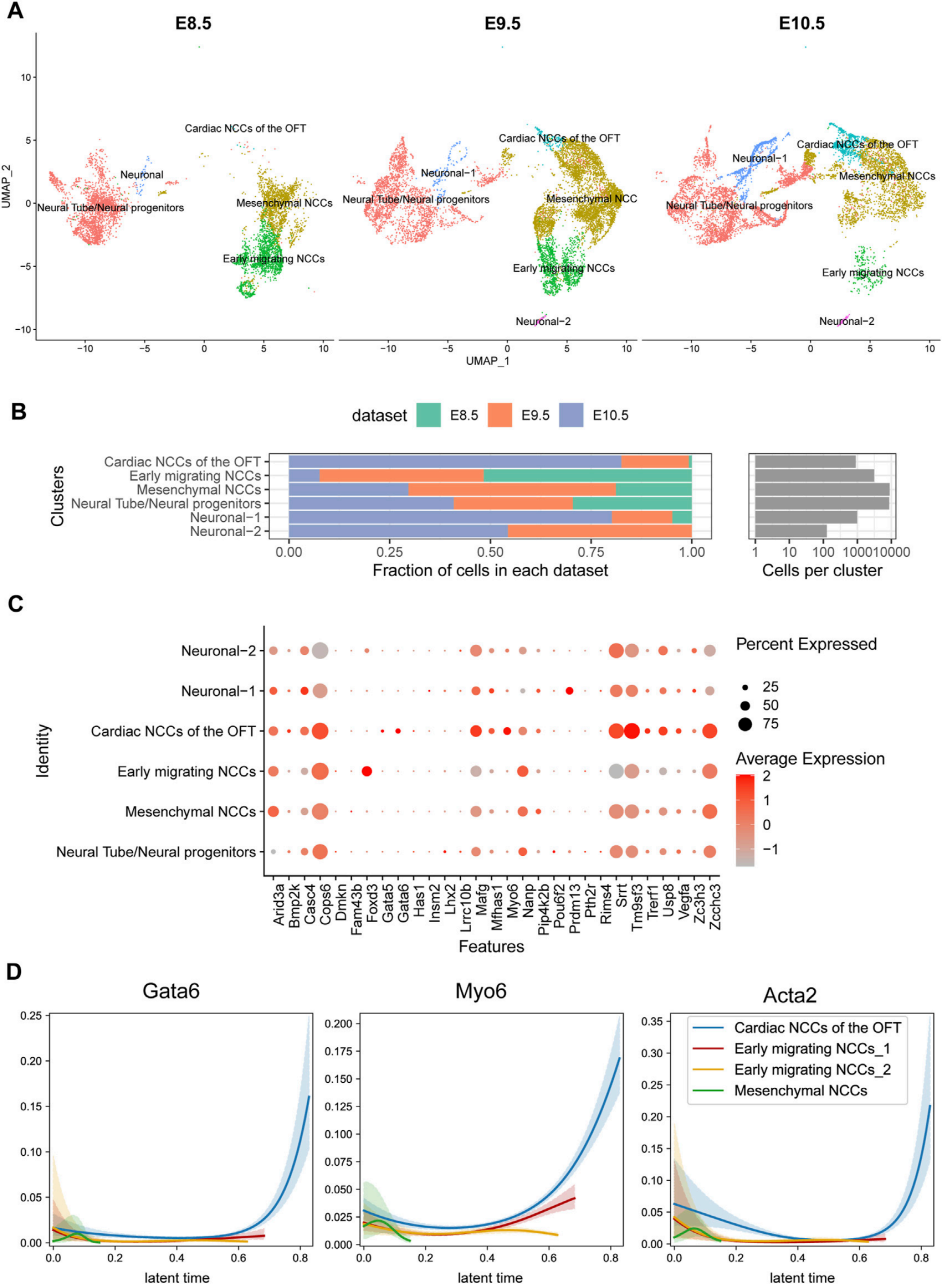
Temporal analysis of single-cell transcriptomics of cardiac neural crest cells in mouse embryos. **(A)** Single-cell clustering within the pharyngeal region at E8.5, E9.5, and E10.5 time points. **(B)** Proportion statistics of each cell type at each time point. **(C)** Expression patterns of candidate genes across cell populations. **(D)** *Gata6* and *Myo6* expression pattern in CNCCs migrating and differentiation process.

Apart from the endocardial lineage, neural crest cells are another major progenitor cell lineage that populated outflow tract. We analyzed the scRNA-seq specific for neural crest lineage from E8.5 to E10.5 in mouse (De Bono et al., 2023). We found that neural crest cells were consisted of four clusters: early migrated NCC, mesenchymal NCC, Cardiac NCC of OFT and neural progenitors on specific markers (Figure 5A). Early migrated neural crest cells were abundant in E8.5 but declined gradually from E9.5. The mesenchymal NCCs were first increased and then decreased, while the cardiac NCC were gradually increased (Figure 5B). We found *FOXD3* highly expressed in the early migrated NCC but nearly diminished in other lineages, indicating *FOXD3* plays a role in the early stage of NCC progenitor differentiation (Figure 5C). *GATA6*, rather than *GATA5*, was also expressed in NCC and increased its expression following NCC development (it was low in early migrated NCC but high in later cardiac NCC) (Figure 5D).

One of 32 candidate genes, *MYO6* is a member of myosin family functioning in a variety of intracellular processes such as vesicular membrane trafficking and cell migration whose expression was also increased during neural crest development. Loss of *MYO6* leads to the deafness in human and animal (Melchionda et al., 2001; Ahmed et al., 2003). A study suggested that *MYO6* mutations in humans were also associated with cardiomyopathies (Hegan et al., 2015). In our cohort, we identified a duplication mutation c.2751dup, resulting in a frameshift change of p.Gln918ThrfsTer24 in 27 LVOTO patients. This mutation, documented as pathogenic in ClinVar, has previously been associated with deafness. However, its association with CHD had not been previously reported. The dynamic expression of *MYO6* in neural crest suggested that a part of OFT malformation may be attributed to functional abnormality of *MYO6* in neural crest.

## Discussion

In this study, we constructed a LVOTO cohort and performed whole exome sequencing to identify 71,430 rare deleterious SNVs and indels in 106 LVOTO patients. Utilizing a gene-based burden test, 32 candidate genes including *GATA5*, *GATA6*, *FOXD3*, *LHX2*, *MYO6* were identified as possibly associated with LVOTO pathogenesis. Among the candidate genes, *GATA5* and *GATA6* have been reported to collaboratively regulate the development of the outflow tract (Laforest and Nemer, 2011; Gharibeh et al., 2018). Interestingly, we identified recurrent variants c.40G>C missense mutation in *GATA5* (20.75%, *n* = 22) and c.993_998 microdeletion in *GATA6* (54.71%, *n* = 58), both of which were enriched in LVOTO patients and not reported in western populations’ LVOTO cohorts. Besides, *VEGFA*, a key gene for vascular development, was identified as another LVOTO candidate gene. In the cohort, we observed 15 LVOTO patients harboring c.329G>T missense mutations. The recurrence of these mutations in our cohort suggests that they may be the founder mutations in the Han Chinese LVOTO population.

Apart from novel SNVs, our study also identified the novel candidate genes *FOXD3* and *LHX2*. Although these genes had not been previously reported to associate with LVOTO, they have been implicated in neural crest and pharyngeal mesoderm development (Stewart et al., 2006; Harel et al., 2012). The identification of mutant genes in neural crest also has a key role in OFT development, which cannot be revealed by lineage tracing approach as in mouse. Additionally, we identified *MYO6* as LVOTO genes that was previously reported to be associated with artery disease (Slavin et al., 2011). Of note, we identified a high impact frameshift mutation c.2751dup in *MYO6* in 31 LVOTO patients, a variant that is associated with deafness (Kwon et al., 2014; Garcia-Garcia et al., 2020). However, there were no deafness observed in these 31 patients. This implies the incomplete penetrance of *MYO6* in deafness onset, or possibly cooperated with distinct genetic modifiers to cause deafness or CHD respectively. Further investigations are required to clarify the underlying mechanism. It is noteworthy that despite the occurrence of incomplete penetrance in disease association analysis, the gene-based burden testing approach utilized in our study has demonstrated robustness, even in the face of diseases with moderate incomplete penetrance (Guo et al., 2016). This underscores the potential of this approach in uncovering genetic contributors to complex disorders, paving the way for more comprehensive genetic studies that could further elucidate the intricate mechanisms underlying LVOTO and similar conditions.

By scRNA-seq, we found LVOTO genes dynamically expressed mainly in two lineages. In the endocardium-derived lineage, *GATA5* and *GATA6* expressed in the early stage and then quickly declined during the process of EndMT. In the cardiac neural crest cell lineage, *GATA6*, *MYO6*, and *FOXD3* were progressively upregulated along with the migration and differentiation of neural crest cells. Moreover, an elevation in *LHX2* expression was observed only in an early progenitors stage. These results illuminate the dynamic transcriptional landscape of candidate genes during OFT development and underscore their distinct functions. This also highlights the complexity of OFT malformation and LVOTO pathogenesis.

*NOTCH1* is a well-established risk gene for LVOTO. However, our burden test did not identify the significant SNV enrichment of *NOTCH1* and other seven previously reported risk genes (Supplementary Figure S3). A reasonable explanation might be that, as a complex disease, the pathogenesis of LVOTO is not solely attributed to a single gene. *NOTCH1*, as the most extensively studied gene associated with LVOTO, accounts for only 6% of patients with LVOT malformations (Roifman et al., 2021). The relatively small size of our cohort or geographical bias of patient enrollment could also influence these findings. A smaller cohort size often leads to reduced statistical power, which can limit our ability to detect and accurately interpret genetic associations and trends. Especially for gene-based burden analyses, analyzing dominant disorders with high locus heterogeneity typically requires larger cohort sizes (Guo et al., 2016). Therefore, future studies should aim to include larger cohorts, which would provide more robust statistical analyses. Besides, the genetic diversity between our Han Chinese cohort and the Western population could be another reason for the failed identification of *NOTCH1* as significantly enriched. Additionally, it is important to mention that there are another 26 genes identified by our association test that may contribute to LVOTO, but were not discussed in detail within this paper. For example, a recent study highlighted an association of *BMP2K* to OFT development (Dai et al., 2013). One study of our group demonstrated *MAFG* as a key regulatory factor that plays a crucial role in the modulation of *VEGFA* expression and angiogenesis (Wang et al., 2019). These genes could potentially possess crucial information to understand the genetic landscape of LVOTO. Moreover, our results suggested LVOTO has a complex genetic architecture and cooperative gene function manner (Figure 2B), therefore, it will become particularly important to clarify the collective gene interacting network for sake of ultimately understanding the genetic pathogenesis of LVOTO.

In sum, our study provides a comprehensive investigation of the genetic architecture of LVOTO in Han Chinese population and new insights into the potential molecular mechanisms underlying the pathogenesis of outflow tract malformations. Our study identified new rare deleterious variants and genes possibly contributing to LVOTO pathogenesis. Further studies are warranted to validate these SNVs and genes and elucidate the underlying molecular mechanisms.

## Data Availability

The original contributions presented in the study are included in the article/Supplementary Material, further inquiries can be directed to the corresponding authors.
