# Supplementary figures and images for: Whole exome sequencing reveals genetic landscape associated with left ventricular outflow tract obstruction in Chinese Han population
Source: Front Genet. 2023 Dec 18;14:1267368. doi: 10.3389/fgene.2023.1267368 (PMC10757952; doi:10.3389/fgene.2023.1267368)

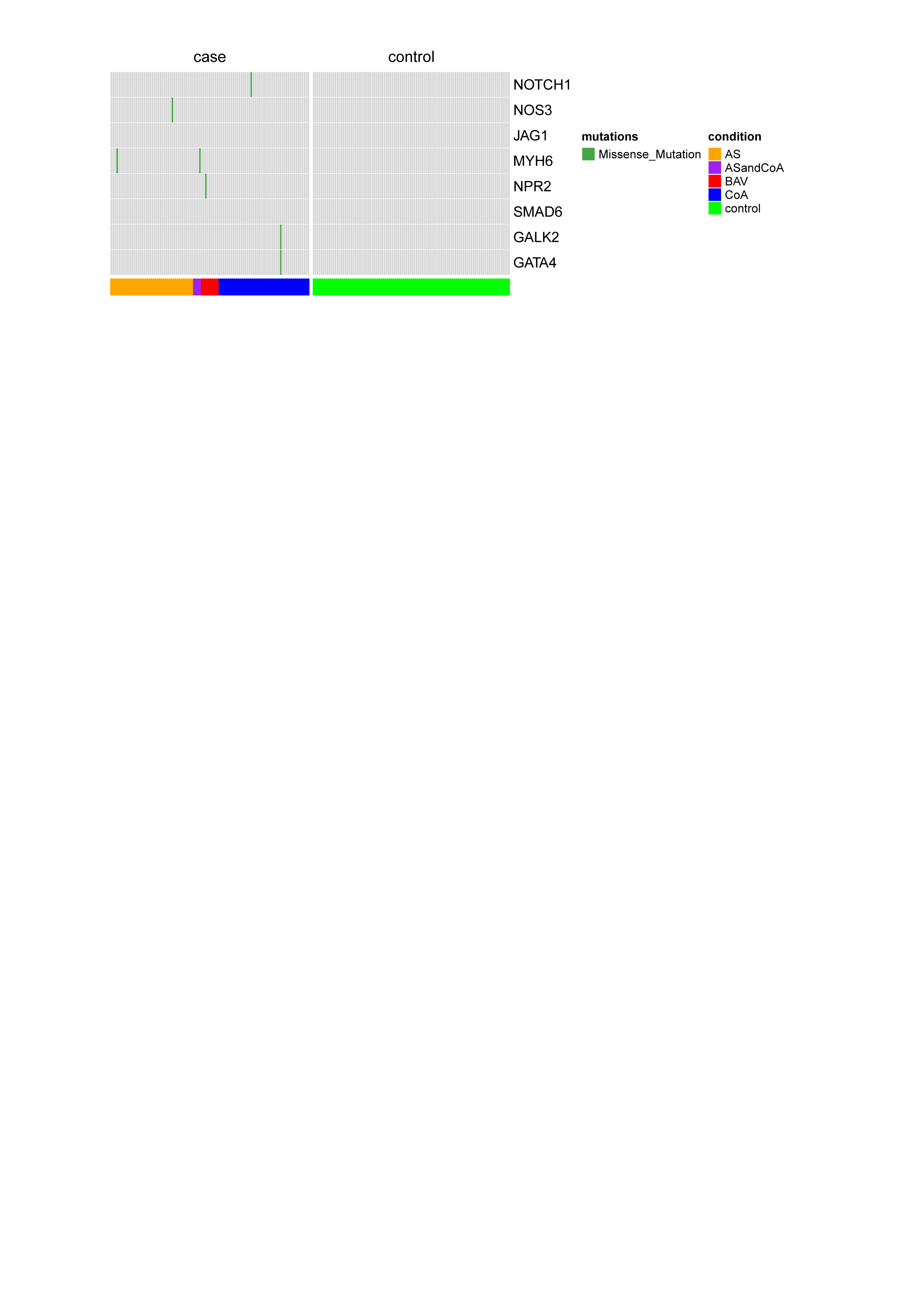

Supplement: Supplementary file 3 [file Image3.TIF]

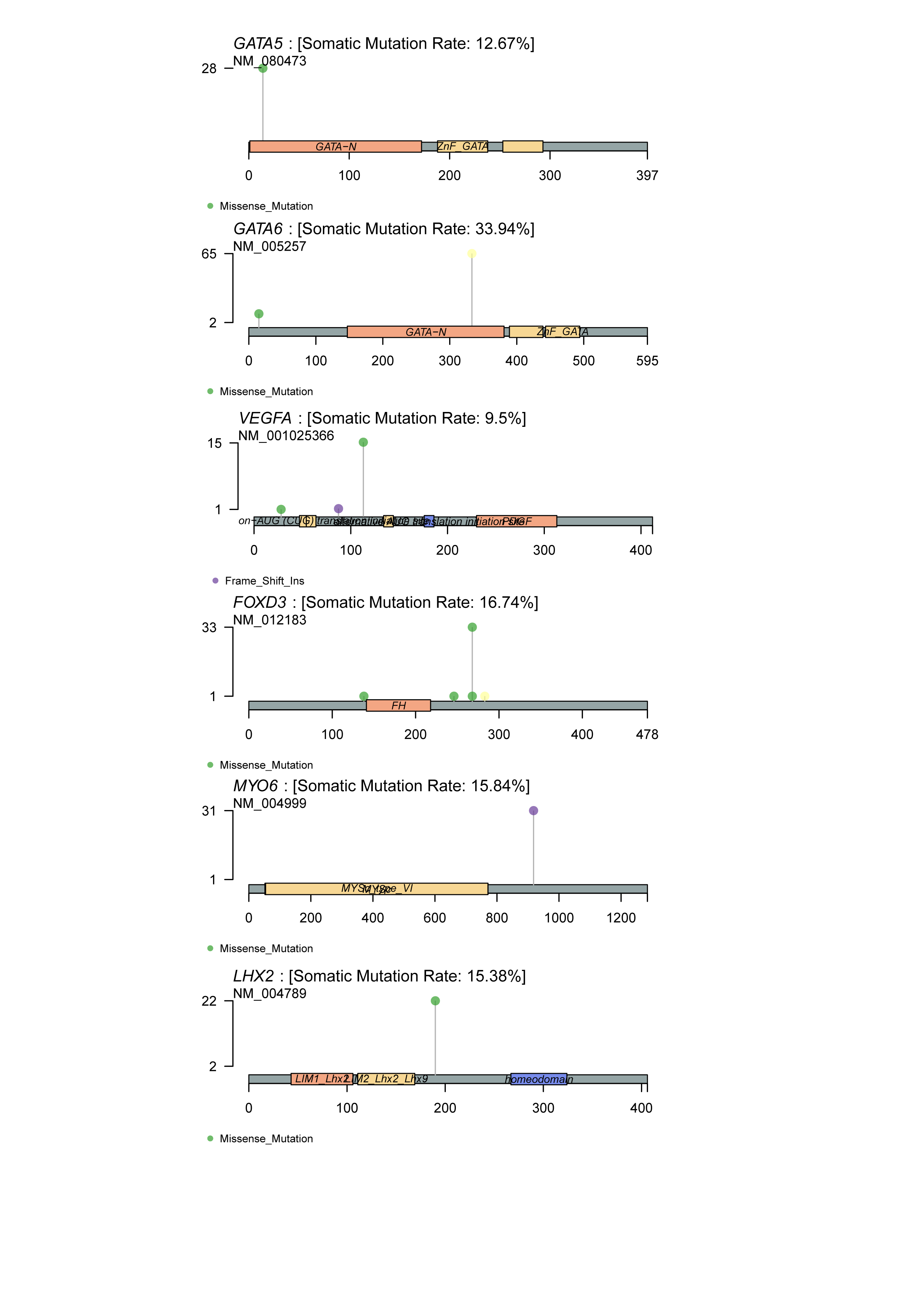

Supplement: Supplementary file 4 [file Image2.TIF]

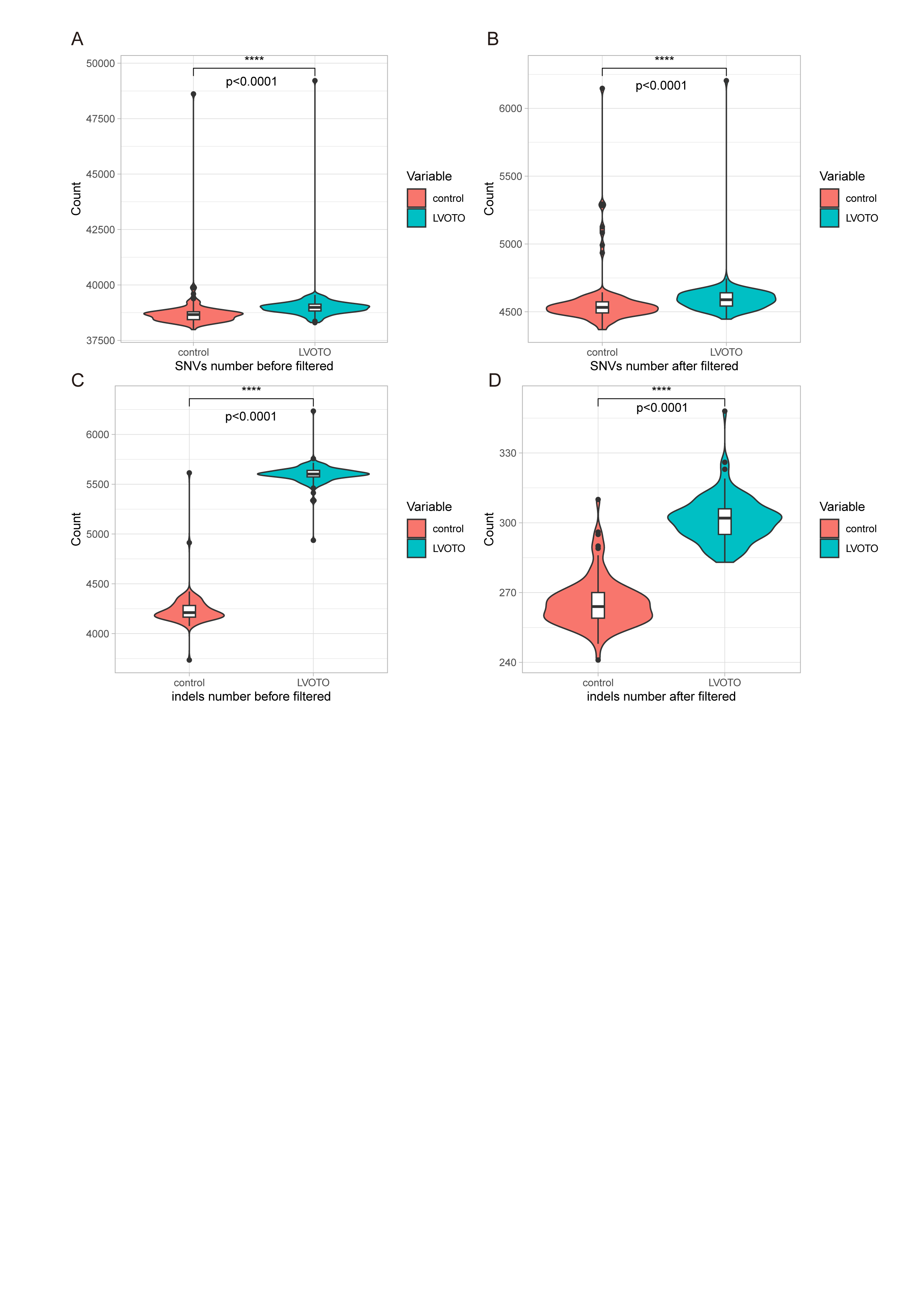

Supplement: Supplementary file 5 [file Image1.TIF]
